# INMI1 Zika Virus NS4B Antagonizes the Interferon Signaling by Suppressing STAT1 Phosphorylation

**DOI:** 10.3390/v13122448

**Published:** 2021-12-06

**Authors:** Elisa Fanunza, Nicole Grandi, Marina Quartu, Fabrizio Carletti, Laura Ermellino, Jessica Milia, Angela Corona, Maria Rosaria Capobianchi, Giuseppe Ippolito, Enzo Tramontano

**Affiliations:** 1 Department of Life and Environmental Sciences, University of Cagliari, 09042 Monserrato, Italy; elisafanunza@live.it (E.F.); nicole.grandi@unica.it (N.G.); lauraerm94@gmail.com (L.E.); jessicamilia47@gmail.com (J.M.); angela.corona@unica.it (A.C.); 2 Department of Biomedical Sciences, University of Cagliari, 09042 Monserrato, Italy; quartu@unica.it; 3 National Institute for Infectious Diseases, L. Spallanzani Scientific Institute for Research, Hospitalization and Healthcare (IRCCS), 00149 Rome, Italy; fabrizio.carletti@inmi.it (F.C.); maria.capobianchi@inmi.it (M.R.C.); giuseppe.ippolito@inmi.it (G.I.)

**Keywords:** Zika virus, interferon, interferon evasion, NS4B, STAT1, STAT2, innate immunity, phosphorylation

## Abstract

The evasion of the Interferon response has important implications in Zika virus (ZIKV) disease. Mutations in ZIKV viral protein NS4B, associated with modulation of the interferon (IFN) system, have been linked to increased pathogenicity in animal models. In this study, we unravel ZIKV NS4B as antagonist of the IFN signaling cascade. Firstly, we reported the genomic characterization of NS4B isolated from a strain of the 2016 outbreak, ZIKV Brazil/2016/INMI1, and we predicted its membrane topology. Secondly, we analyzed its phylogenetic correlation with other flaviviruses, finding a high similarity with dengue virus 2 (DEN2) strains; in particular, the highest conservation was found when NS4B was aligned with the IFN inhibitory domain of DEN2 NS4B. Hence, we asked whether ZIKV NS4B was also able to inhibit the IFN signaling cascade, as reported for DEN2 NS4B. Our results showed that ZIKV NS4B was able to strongly inhibit the IFN stimulated response element and the IFN-γ-activated site transcription, blocking IFN-I/-II responses. mRNA expression levels of the IFN stimulated genes *ISG15* and *OAS1* were also strongly reduced in presence of NS4B. We found that the viral protein was acting by suppressing the STAT1 phosphorylation and consequently blocking the nuclear transport of both STAT1 and STAT2.

## 1. Introduction

ZIKV is a mosquito-borne virus, belonging to the family of *Flaviviridae*, causing a flu-like illness associated with neurological complications [1]. After the 2015–2016 outbreak, ZIKV is continuing to spread, remaining a global public health threat, for which no approved vaccines or antivirals are yet available [2]. ZIKV is characterized by a positive-sense, single-stranded RNA genome (ssRNA+), encoding a polyprotein that is processed into three structural proteins, the capsid (C), membrane (prM) and envelope (E) and seven non-structural (NS) proteins (NS1, NS2A, NS2B, NS3, NS4A, NS4B and NS5) [3].

The viral protein NS4B is a highly hydrophobic protein with a molecular weight of about 27 kDa, integrated into the endoplasmic reticulum membrane. NS4B is a component of the viral replication complex [4,5]. In addition, it mediates virus–host interactions by suppressing the host RNA interference [6] and evading the innate interferon (IFN) response [7].

The NS4B antagonism of the IFN system has critical implications in ZIKV immune pathogenesis and virulence [8]. It has been demonstrated that a single mutation in NS4B, associated with a decreased IFNβ production and diminished expression of the IFN stimulated genes (ISGs), causes increased viral replication and neurovirulence in mice, confirming the importance of NS4B as a determinant of pathogenesis [9].

NS4B is a well-known IFN inhibitor in different flaviviruses, mediating the evasion of both IFN production and signaling [7]. In fact, on one hand, yellow fever virus (YFV), hepatitis C virus (HCV), duck Tembusu virus (TMUV) and bovine viral diarrhea virus (BVDV) NS4B have been reported to block the RIG-I /MDA-5 pathway of IFN production [10,11,12,13,14]. On the other hand, dengue virus (DENV), West Nile virus (WNV) and YFV NS4B were shown to inhibit the IFN signaling pathway by suppressing the phosphorylation and nuclear transport of STAT1/STAT2 [15,16,17,18]. In particular, the IFN signaling inhibitory effect was reported for DEN2, WNV9/NY99, Kunjin virus (KUNV) and YFV1 NS4B, also highlighting that IFN evasion mechanisms may differ between flaviviruses [7]. 

Strain-specific differences characterize also the IFN inhibition mediated by ZIKV NS4B. Recent studies have shown the ability of NS4B strains FSS13025, Z1106033, Puerto Rico-PRVABC-59 and Dakar-41525 to inhibit the RIG-I pathway activation through blocking TBK1 phosphorylation [19,20,21]. While the evasion of the IFN production cascade has been well characterized, the IFN signaling inhibition, mediated by NS4B, has not been investigated yet.

In this study, we explored the role of NS4B as an antagonist of the IFN signaling cascade. We based our analysis on the sequence of the isolate ZIKV Brazil/2016/INMI1 (also referred to as ZIKV INMI1), which has been previously reported to not induce either type I or type III IFN, slightly activating only type II IFN in PBMC, and thus involving a mechanism of IFN evasion [22]. In a previous study, we analyzed the ability of viral proteins, encoded by the ZIKV INMI1 strain, to block the IFN system, and found that the expression of NS2A resulted in robust IFN signaling inhibition [23]. In the present study, we found that overexpression of NS4B also inhibits the signaling of both types I and II IFNs, mediating the block of STAT1 phosphorylation and the nuclear transport of both STAT1 and STAT2, as already reported for other flaviviruses. This novel NS4B mechanism provides additional insights for the study of the IFN evasion mediated by ZIKV.

## 2. Materials and Methods

### 2.1. Cells and Reagents

HEK293T or Vero E6 (American Type Culture Collection, USA) were grown at 37 °C in a 5% CO_2_ humidified incubator. Cells culture media was Dulbecco’s modified Eagle’s medium (Gibco, Waltham, MA, USA) with supplementation of 10% fetal bovine serum (Gibco, Waltham, MA, USA) and 1% penicillin-streptomycin (Sigma, St. Louis, MO, USA). Plasmid pNFκB-luc was kindly given by Prof Ian Goodfellow (University of Cambridge, Cambridge, UK). IFN-α, IFN-γ, TNF-α and Pierce ECL Western blotting substrate were from Thermo Fisher Scientific (Waltham, MA, USA).

### 2.2. Virus Stock Preparation

Vero E6 cells were infected with ZIKV 2016/INMI1 isolate (GenBank Accession number KU991811), obtained from the serum of a traveler returning from Brazil to Italy. Cell lysates were clarified, aliquoted and stored at −80 °C until use. A limiting dilution assay was performed on Vero E6 cells for titering the virus. Viral titers (10^6.12^ TCID_50_/mL) were measured with the method of Reed and Muench.

### 2.3. Construction of 2K-NS4B Expression Plasmid

2K-NS4B gene was amplified from the Brazil/2016/INMI1 isolate. The coding sequence with a C-terminal FLAG was subcloned into the pcDNA3.1 (+) using NheI and EcoRI. The primer pairs used for subcloning are listed in Table 1.

### 2.4. Luciferase Reporter Gene Assays

Dual luciferase assays were previously described in [24,25]. Briefly, HEK293T or Vero cells (1.5 × 10^4^ cells/well) were transfected with plasmids pIFNβ-luc, pISRE-luc, pGAS-luc or NF-kB-luc to quantify IFN-β production, IFN-I, IFN-II or NF-kB signaling, respectively. After stimulation of cells with Influenza Virus (IAV) RNA (333 ng/mL), IFN-α (10 ng/mL), IFN-γ (333 ng/mL) or TNF-α (40 ng/well) to induce the relative pathways, cells were harvested for luminescence analysis.

### 2.5. EC_50_ and FIC_50_ Calculations

GraphPad Prism software was used to calculate the EC_50_ of ZIKV NS2A and NS4B, based on three independent experiments (each performed in triplicate). The fractional inhibitory concentrations (FIC) of the respective proteins (A: NS2A; B: NS4B) were calculated with Equations (1)–(3) and determined based on the following definitions: FIC_50_ < 1, synergistic action; FIC_50_ = 1, additive action; FIC_50_ > 1, antagonistic action [26,27].

FIC_50_ A = [EC_50_ (A + B)]/EC_50_ A (1)

FIC_50_ B = [EC_50_ (B + A)]/EC_50_ B (2)

FIC_50_ = FIC_50_ A + FIC_50_ B (3)

### 2.6. Immunoprecipitation

Immunoprecipitation of FLAG-NS2A or FLAG-NS4B was performed as previously described [28]. Membranes were probed with mouse monoclonal anti-FLAG M2 (1 μg/mL) (Sigma, St. Louis, MO, USA) and rabbit polyclonal anti-GAPDH (1:1000) (Cell Signaling, Denver, MA, USA) was used as primary antibodies. Goat anti-mouse (1:10,000) and goat anti-rabbit (1:2000) HRP-linked IgG (Cell Signaling, Denver, MA, USA) were used as secondary antibodies. Images were captured with Chemidoc MP Imaging System (Bio-Rad, Hercules, CA, USA).

### 2.7. Immunoblot Analysis

HEK293T cells (4 × 10^5^ cell/mL) were transfected with empty vector or ZIKV FLAG-NS4B plasmid for 36 h, then cells were stimulated with IFN-α (10 ng/mL) for 30 min. Cell lysates were analyzed by Western blot [23]. Membranes were probed with monoclonal rabbit primary antibodies against P-STAT1 (1:1000) and GAPDH (1:1000) (Cell Signaling, Denver, MA, USA), and polyclonal rabbit primary antibody against STAT1-tot (1:1000) (Cell Signaling, Denver, MA, USA). The secondary antibody was goat anti-rabbit HRP-linked IgG (1:2000) (Cell Signaling, Denver, MA, USA). Detection was performed using Pierce ECL Western blotting substrate and Chemidoc MP Imaging System (Biorad, Hercules, CA, USA).

### 2.8. Immunofluorescence

HEK293T cells (3 × 10^5^ cell/well) were transfected with empty vector or plasmids for ZIKV proteins. Stimulation with IFN-α (10 ng/mL) was performed after 24 h or 36 h of transfection, then cells were subjected to immunofluorescence. Cells were incubated with the monoclonal primary antibodies anti-Flag M2 produced in mouse (1:500) (Sigma, St. Louis, MO, USA) and anti-P-STAT1 (1:400) (Cell Signaling, Denver, MA, USA) or anti-P-STAT2 (1:200) (Cell Signaling, Denver, MA, USA) produced in rabbit for 1 h at room temperature. Then, cells were incubated with secondary antibodies goat anti-mouse conjugated to Alexa Fluor 488 (1:500) (Cell Signaling, Denver, MA, USA) and goat anti-rabbit conjugated to Alexa Fluor 594 (1:500) (Cell Signaling, Denver, MA, USA) for 1 h at room temperature. Nuclei were stained with Hoechst (Thermo Fisher Scientific, Waltham, MA, USA). 1,4-Diazabicyclo(2,2,2)octane (DABCO) was used as anti-fade reagent. Images were captured at 40× magnification. Cell Counter plugin image analysis program ImageJ was used for cell count.

### 2.9. RNA Extraction and Quantitative Real-Time PCR

HEK293T (3 × 10^5^ cell/well) were transfected with empty vector or expression plasmids for ZIKV proteins for 36 h. Then, cells were stimulated with IFN-α (10 ng/mL) for 24 h. RNA extraction was performed as previously reported [29]. Quantitative real-time PCR (RT-qPCR) was performed using Luna Universal One-Step RT-qPCR kit (New England Biolabs, Ipswich, MA, USA), using primers in Table 2. GAPDH was used for normalization. Folds induction of stimulated over not stimulated samples were shown in the graphs.

### 2.10. Bioinformatics

#### 2.10.1. NS4B Topology Prediction

The amino acid sequence of 2K-NS4B (strain Brazil/2016/INMI1) was used for membrane topology prediction using the following webservers: HMMTOP (http://www.enzim.hu/hmmtop/index.php (accessed on 9 June 2020)), DAS (https://tmdas.bioinfo.se/DAS/index.html (accessed on 9 June 2020)), TMHMM2 (http://www.cbs.dtu.dk/services/TMHMM/ (accessed on 9 June 2020)), TMpred (https://embnet.vital-it.ch/software/TMPRED_form.html (accessed on 9 June 2020)), TOPCONS (http://topcons.cbr.su.se/ (accessed on 9 June 2020)) and Split (http://split.pmfst.hr/split/4 (accessed on 9 June 2020)).

#### 2.10.2. Alignments

Geneious version 8.1.4 [30] and MAFFT algorithm FFT-NS-i x1000 [31] were used for designing NS4B primers, using amino acids sequences of INMI1, MR766, Ganxian, DENV-1, YFV and WNV [23].

Clustal Omega [32] was used to calculate percent identity among INMI1 NS4B and other flaviviruses.

#### 2.10.3. Phylogenetic Analysis

A neighbor joining analysis was performed to build the NS4B phylogenetic tree [23], using amino acid sequences from Uniprot database (https://www.uniprot.org (accessed on 23 June 2020)). Mega Software version 6 [33] was used for multiple alignments.

### 2.11. Graphics

GraphPad Prism software 6.01 (GraphPad software, Inc, San Diego, CA, USA, 2012) was used to perform graphics of experiments.

## 3. Results

### 3.1. NS4B Genomic Characterization and Prediction of Membrane Topology

**Figure 1:**
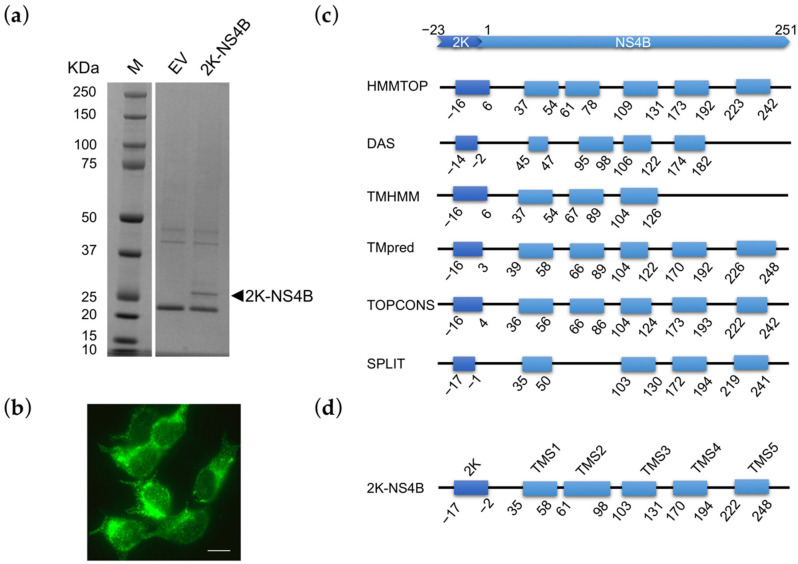
ZIKV INMI1 NS4B expression and prediction of membrane topology. Protein expression level was verified by (**a**) SDS-Page and (**b**) immunofluorescence; scale bar, 10 μm; (**c**) schematic representation of ZIKV INMI1 NS4B transmembrane segments predicted by HMMTOP, DAS, TMHMM, TMpred, TOPCONS and Split; the blue boxes represent predicted transmembrane segments (TMS); the positions of the first and last amino acid of TMS are indicated; (**d**) consensus model of ZIKV INMI1 NS4B topology.

The infectious strain ZIKV INMI1 was isolated from the serum of a traveler returning to Italy from Brazil in 2016. The whole genome was amplified, sequenced and submitted to GenBank (KU991811.1), resulting in a stretch of 10643 nucleotides (nt) encoding a large polypeptide, containing 3423 amino acids. ZIKV INMI1 NS4B protein sequence was predicted from alignment with NS4B proteins of MR766 ZIKV, Ganxian ZIKV, DENV, YFV and WNV, and considering as reference sequence for ZIKV a consensus sequence obtained from the alignment of 9 African lineage strains and 25 Asiatic lineages [34]. Based on our alignment, we found a 69 nt region upstream the NS4B start site which is conserved between ZIKV INMI1 and other flaviviruses and that corresponds to a 2-kDa signal peptide (2K) of 23 amino acids [35]. ZIKV INMI1 2K-NS4B sequence consists of 822 nt, coding for a 274 residues protein, with a predicted weight of about 29 KDa (including the 2K peptide). We generated a plasmid encoding the ZIKV 2K-NS4B C-terminally fused to a FLAG epitope. We co-transfected HEK293T cells with an empty vector (EV) or with the construct 2K-NS4B-FLAG and we confirmed the molecular weight of 2K-NS4B by SDS-PAGE of the immunoprecipitated protein (Figure 1a).

Expression of the protein was also verified by immunofluorescence, which allowed to observe a cytoplasmic staining with a distinct reticular pattern, particularly concentrated in the perinuclear region (Figure 1b). Flaviviral NS4B is an integral membrane protein. Its membrane topology has been proposed using different structural analysis methods [36,37,38]. The N-terminal portion contains three hydrophobic segments (including the 2K signal peptide) that interact with the lumen side of the endoplasmic reticulum (ER) membrane; the C-terminal end contains three transmembrane segments [36]. To establish a membrane topology model of ZIKV INMI1 NS4B we compared predictions of trans-membrane segments (TMSs), obtained with different algorithms (Figure 1c). TMSs predictions by the different algorithms were substantially concordant, yielding the segment boundaries at approximately the same positions for almost all programs used. In the consensus topology (Figure 1d), the 2K peptide was predicted to be transmembrane; two and three TMSs were predicted to be at the NS4B N-and C-terminal parts, respectively. This model is consistent with that of other flaviviruses, such as YFV [39], and with the experimentally determined topology of DENV NS4B protein [36].

### 3.2. Sequence and Phylogenic Analysis of NS4B

**Figure 2:**
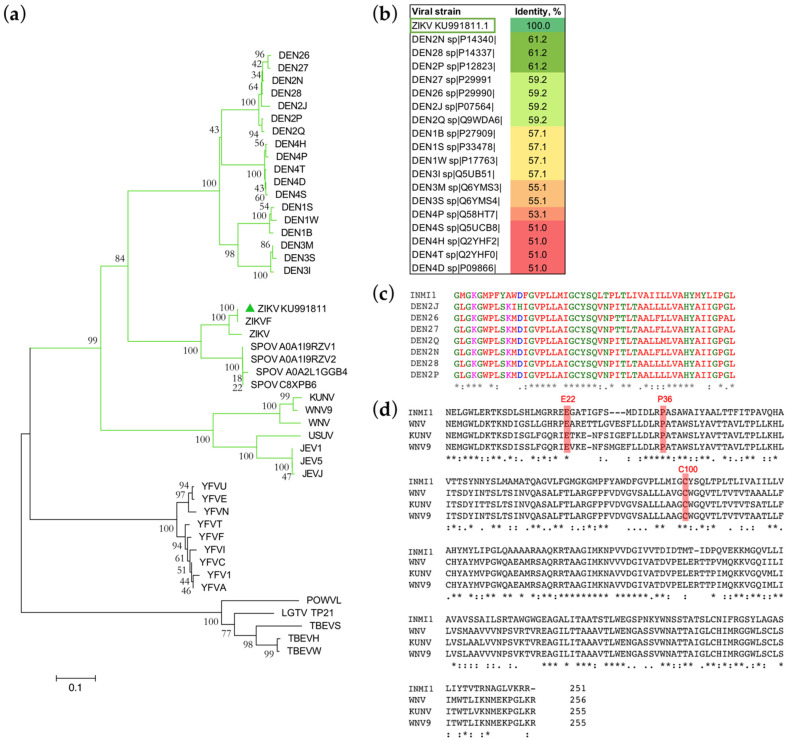
Phylogenic analysis of NS4B. (**a**) Phylogenetic tree of the amino acid sequence of NS4B protein of ZIKV INMI1 and of other representative flaviviruses. The highly supported cluster formed by the Spondweni (SPOV) group of mosquito-borne flaviviruses, including INMI1 KU991811 (green triangle) and other ZIKV strains, is highlighted with green branches; (**b**) amino acid identity matrix of NS4B_77-125_; (**c**) amino acid alignment of NS4B_77-125_ of INMI1 KU991811 and DEN2 isolates; (**d**) amino acid alignment of INMI1 KU991811 NS4B and WNV, KUNV and WNV9 whole NS4B; residues important for IFN modulation in WNV are highlighted in red; (*) identical nucleotides, (:) conserved substitution, (.) semi-conserved substitution.

To understand the phylogeny of ZIKV INMI1 NS4B, the deduced amino acid sequence was aligned with those of representative flaviviruses with available sequence data. An NJ tree was generated, and bootstrap resampling with 1000 replicates was employed to place approximate confidence limits on individual branches (Figure 2a).

A close relationship between the ZIKV INMI1 NS4B and other ZIKV isolates was found with a 100% of bootstrap value (Figure 2a). The high correlation of INMI1 ZIKV NS4B with other ZIKV strains NS4B was also confirmed by percentage identities between 96% and 100% (Figure S1a,b). Belonging to the Spondweni (SPOV) group of mosquito-borne flaviviruses [40], NS4B phylogenetic tree showed a high statistical correlation with different strains of SPOV (Figure 2a). Moreover, the alignment of flaviviral NS4B resulted in up to 82% similarity between amino acid sequences of NS4B ZIKV and SPOV (Figure S2a). In addition to SPOV, DENV is the viral species most closely related to ZIKV. In fact, we also observed high statistical correlation with DENV NS4B, confirmed by the high bootstrap values (Figure 2a). Notably, among DENV isolates, the highest percentage identity was found with DEN2 strains (Figure S2a). The IFN signaling inhibitory mediated by NS4B of DEN2 has been previously investigated [15]. In particular, the first 125 amino acids of DEN2 NS4B are reported to be sufficient for inhibition of α/β interferon signaling [16]. Hence, we aligned INMI1 NS4B_1-125_ with NS4B_1-125_ of the four DENV strains. Results showed that the highest identity percentages were found between INMI1 and DEN2 isolates (Figure S2b). More in-depth analysis, carried out by Muñoz et al. [16], showed that amino acid residues located between amino acids 77 and 125 in DEN2 NS4B play an essential role in the inhibition of IFN signaling [16]. To evaluate whether these amino acids were conserved in ZIKV, we aligned ZIKV INMI1 NS4B_77-125_ with DENV NS4B_77-125_. Notably, we found that this specific region was also highly conserved in ZIKV, showing up to 61% identity with DEN2 strains (Figure 2b,c), possibly indicating that such region might have a functional role in the NS4B IFN signaling inhibition, which should be further evaluated.

It has also been demonstrated that in NS4B of WNV amino acids in the N-terminal domain are involved in the modulation of the IFN response [41]. In KUNV residues E22 and K24 were found to be critical for inhibition of STAT1 phosphorylation [18] and were reported to control IFN resistance in cells expressing sub-genomic replicons [41]. Furthermore, mutations in WNV NS4B residues P38 and C102 were reported to induce lower levels of viremia in mice relative to wild-type NS4B [42] and led to high IFN-I response in WNV [42,43]. We compared the WNV and ZIKV INMI1 sequences to identify whether residues associated with IFN modulation were also present in the newly sequenced ZIKV INMI1 NS4B. Despite the moderate identity percentages observed for WNV (42.4%), WNV9 (43.4%) and KUNV (43%) (Figure S2a), amino acid residues involved in the IFN response in WNV were conserved in ZIKV INMI1 NS4B (E22, P36 and C100, corresponding to WNV E22, P38 and C102, respectively) (Figure 2d). Taken together, these data support the hypothesis of a NS4B function in IFN signaling inhibition.

### 3.3. NS4B Inhibits IFN-β Production and Type I and II IFN Signaling

**Figure 3:**
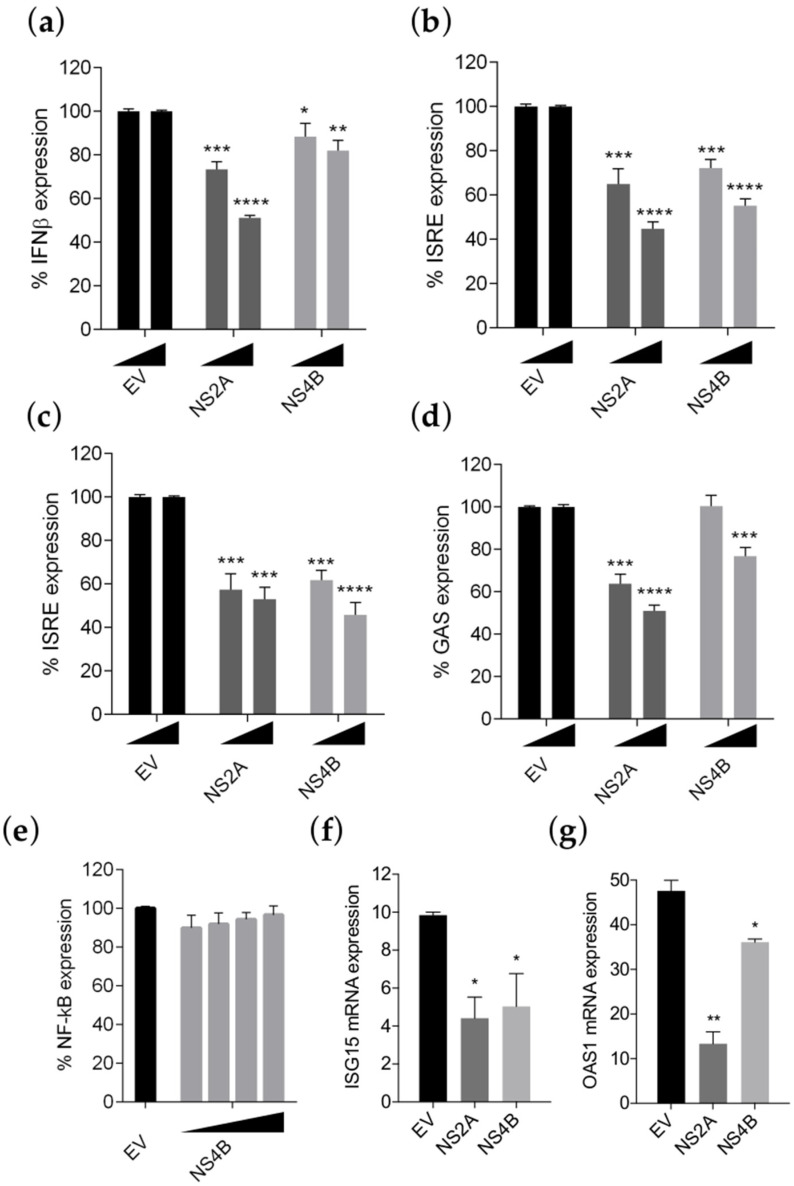
ZIKV INMI1 NS4B inhibits IFN production and signaling. (**a**) Percentage of pIFNβ-luc expression in HEK293T cells transfected with increasing concentrations (30 ng/well and 60 ng/well) of empty vector (EV), ZIKV NS2A or NS4B after stimulation with IAV vRNA; percentage of pISRE-luc expression in (**b**) HEK293T cells and (**c**) Vero cells transfected with increasing concentrations (30 ng/well and 60 ng/well) of EV, ZIKV NS2A or NS4B and stimulated with IFN-α; (**d**) percentage of pGAS-luc expression in HEK293T cells transfected with increasing concentrations of EV, ZIKV NS2A or NS4B after stimulation with IFN-γ; (**e**) percentage of NF-κB expression in HEK293T cells transfected with increasing concentrations of ZIKV NS4B (7.5–15–30–60 ng/well) after stimulation with TNF-α; (**f**) *ISG15* and (**g**) *OAS1* transcript levels in HEK293T cells transfected with EV, ZIKV NS2A or NS4B in presence of IFN-α. (**a**–**e**) Results are shown as percentage of (**a**) pIFN-β-luc; (**b**,**c**) pISRE-luc; (**d**) pGAS-luc and (**e**) pNFκB-luc activation in NS2A or NS4B transfected cells over empty vector (EV) transfected controls (100%). (**a**–**g**) Significance calculated using a two-tailed unpaired Student’s *t*-test, * *p*< 0.05, ** *p* <  0.01, *** *p* <  0.001, **** *p* <  0.0001. Error bars indicate the mean ± SD (data from at least 3 independent experiments).

To investigate if effectively ZIKV INMI1 NS4B has a role in evading the IFN response in ZIKV, we evaluated its effect using two previously developed dual-luciferase cell-based assays, able to quantify the IFN production and the IFN signaling and their inhibition by viral proteins [24,25]. Recent studies demonstrated that NS4B of FSS13025, Z1106033, PRVABC-59 and Dakar-41525 ZIKV strains were able to antagonize the IFN-β promoter activation [20,44]. Since all of these strains showed >96% amino acid identity with the ZIKV INMI1 strain (Figure S1b), we aimed to verify if also ZIKV INMI1 NS4B was able to suppress the IFN-β production, we co-transfected HEK293T cells with IFNβ-luc promoter, driven by the firefly luciferase, and a renilla luciferase vector (pRL-TK), as control of transfection efficiency [24], with the expression plasmid for ZIKV INMI1 NS4B, at two different concentrations. We used ZIKV NS2A as positive control of inhibition [23]. As expected, both proteins were able to inhibit the IFN production pathway, even though NS2A seemed to be more effective than NS4B (Figure 3a).

Beside antagonizing the production of IFN-β, it has been previously demonstrated that NS4B proteins efficiently block the IFN signaling cascade in different flaviviruses [7]. To address whether also ZIKV NS4B exerts the same function, we co-transfected HEK293T cells with NS4B plasmid and the IFN stimulated response element (ISRE) promoter driven by the firefly luciferase. pRL-TK transfection was used as control for transfection efficiency. After 24 h, co-transfection was followed by stimulation with IFN-α, and luciferase activity was measured. The gene-reporter data indicated that, in cells transfected with NS4B expression plasmid, there was a significant and dose-dependent inhibition of the JAK-STAT signaling (Figure 3b). A similar effect was observed also in Vero E6 cells (Figure 3c).

To investigate whether ZIKV INMI1 NS4B expression also caused inhibition of the IFN-γ response, IFN-γ-induced GAS promoter activation status in NS4B transfected cells, exposed to IFN-γ, was evaluated. Results showed that the IFN-γ-induced GAS promoter expression was significantly inhibited by NS4B (Figure 3d).

To exclude that ZIKV INMI1 NS4B caused a general inhibition of cell-signaling pathways, HEK293T cells were transfected with a plasmid containing an NF-κB responsive promoter driving the expression of the firefly luciferase, pRL-TK and NS4B expression plasmid. After 24 h, cells were incubated with TNF-α, and luciferase activities were determined. We observed that firefly values were not altered by expression of NS4B, indicating that TNF-signaling pathway was not suppressed by this protein, confirming the specific function of NS4B on inhibition of the IFN system (Figure 3e).

Moreover, the expression levels of two human ISGs, *ISG15* and *OAS1*, were determined both in empty vector and in NS4B transfected HEK293T cells receiving IFN-α treatment. Results showed that transcription levels of both *ISG15* and *OAS1* were reduced in NS4B transfected cells compared to the control, and statistical significance was achieved, as measured by RT-PCR (Figure 3f,g). Expression levels of both proteins were verified by immunoprecipitation (Figure S3). Taken together, these results suggest that ZIKV INMI1 NS4B is a potent antagonist of IFN-mediated JAK-STAT signaling.

### 3.4. NS4B Inhibits the STAT1 Phosphorylation

**Figure 4:**
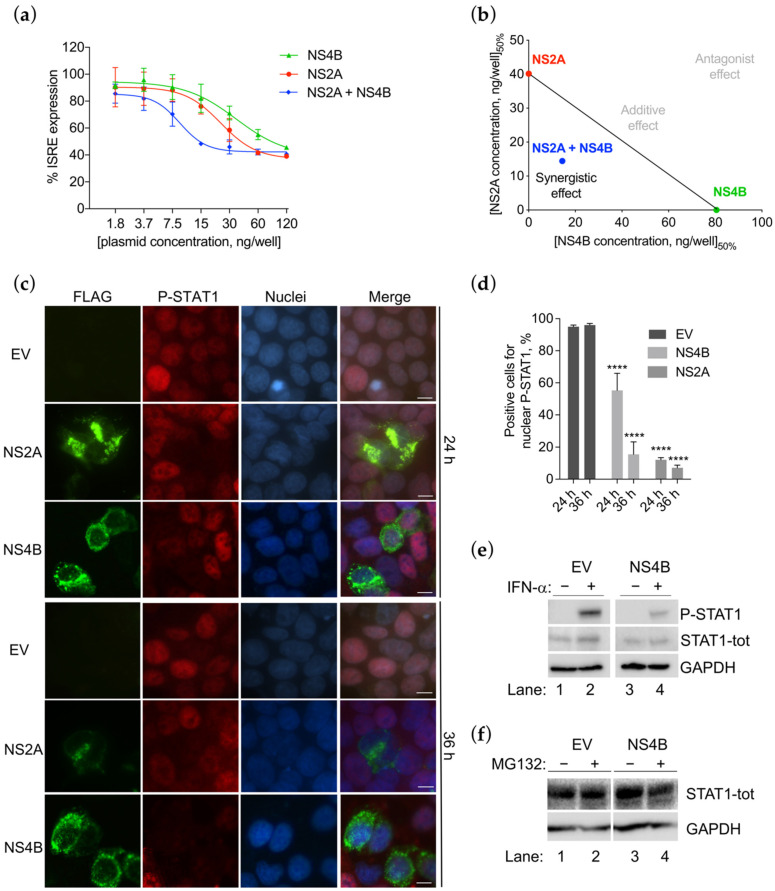
ZIKV INMI1 NS4B inhibits STAT1 phosphorylation. (**a**) Inhibition of pISRE-luc expression in HEK293T cells transfected with increasing concentrations of ZIKV NS2A (red line), NS4B (green line) or the combination of the two plasmids (blue line), and stimulated with IFN-α (10 ng/mL). Results are shown as percentages of pISRE-luc activation in NS2A or NS4B transfected cells over empty vector (EV) transfected controls; (**b**) the isobologram visualizes the synergistic effect of the combination of ZIKV NS2A and NS4B at their respective EC_50_; (**c**) immunofluorescence of HEK293T transfected for 24 h and 36 h with ZIKV NS2A (row 1 and 3) and NS4B (row 2 and 4) and stimulated with IFN-α; FLAG (green) and P-STAT1 (red) signals are detected; nuclei (blue) are stained with Hoechst; scale bar, 10 μm; (**d**) quantification was performed counting positive cells for nuclear P-STAT1 (red signal) in EV, NS2A and NS4B transfected cells, 6–10 fields per each condition, from a total of 3 biologically independent experiments, were analyzed; cells count was performed using Cell Counter plugin of the image analysis program ImageJ, **** *p* <  0.0001 as obtained by unpaired t test. (**e**) Western blot of HEK293T transfected with empty vector (EV) (lane 1–2) or ZIKV NS4B (lane 3–4) stimulated (+) (lanes 2–4) or not (−) (lanes 1–3) with IFN-α; membranes were stained for P-STAT1, STAT1-tot and GAPDH antibodies; (**f**) Western blot of HEK293T transfected with empty vector (EV) (lane 1–2) or ZIKV NS4B (lane 3–4); cells were treated (+) (lanes 2–4) or not (−) (lanes 1–3) with MG132; blot membranes were stained for STAT1-tot and GAPDH antibodies.

We previously reported that transfection with ZIKV INMI1 NS2A resulted in the strong inhibition of IFN-α stimulation of ISRE promoter [23]. In the present study, we performed a dose-response curve co-transfecting cells with increasing concentration of each expression plasmid for the two proteins (1.8–120 ng/well). Calculating the EC_50_ value, we found that NS2A and NS4B were able to dose-dependently suppress the ISRE transcription, showing EC_50_ values of 40.15 ng/well and 93 ng/well, respectively (Figure 4a,b).

Notably, the combined effect of the two proteins resulted in an EC_50_ value of 14.42 ng/well, suggesting that the two proteins were acting synergistically (Figure 4a,b). To further confirm the synergism, the combined effect was represented mathematically via the fractional inhibitory concentration (FIC) index (Table 1). NS2A and NS4B interacted synergistically as the FIC_50_ value was lower than 1 (FIC index = 0.5) (Table 3). 

Subsequently, we aimed to investigate whether the two proteins were acting through the same mechanism of action. We previously reported that ZIKV NS2A inhibits STAT1 phosphorylation [23]. To explore whether NS4B could also prevent the same step, we transfected HEK293T cells with the expression plasmid for FLAG-tagged NS4B for 24 h and 36 h, then we stimulated cells with IFN-α, and analyzed the phosphorylation of STAT1 protein by immunofluorescence assay (Figure 4c). Results showed that NS4B was able to strongly inhibit the STAT1 phosphorylation. The inhibition effect of NS4B was more evident after 36 h of transfection compared with 24 h, with a reduction in P-STAT1 signal of 81% and 45%, respectively (Figure 4d). The reduction in STAT1 phosphorylation was also confirmed by Western blot (Figure 4e). While P-STAT1 was reduced, total STAT1 expression levels were not affected (Figure 4e). To confirm that NS4B was not mediating a STAT1 proteasomal degradation, we asked whether the proteasome inhibitor MG132 could affect the STAT1 levels (Figure 4f). Results showed that STAT1 expression levels did not change in the presence or absence of MG132, confirming that NS4B inhibition was not due to increased STAT1 degradation (Figure 4f).

### 3.5. NS4B Blocks P-STAT2 Nuclear Transport

**Figure 5:**
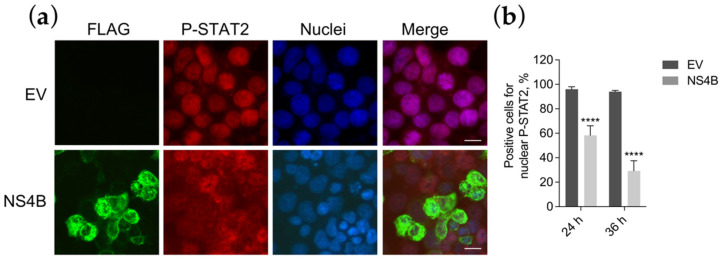
ZIKV INMI1 NS4B blocks STAT2 nuclear transport. (**a**) Immunofluorescence of HEK293T cells transfected with empty vector (EV) or ZIKV NS2A (row 3) stimulated with IFN-α. FLAG (green) and P-STAT2 (red) signals were detected; nuclei (blue) were stained with Hoechst; scale bar, 10 μm; (**b**) quantification was performed counting positive cells for nuclear P-STAT1 (red signal) in EV and NS4B transfected cells; 6–10 fields per each condition, from a total of 3 biologically independent experiments, were analyzed; cells count was performed using Cell Counter plugin of ImageJ. **** *p* <  0.0001 values are reported, as obtained by unpaired *t* test.

It is known that P-STAT1 and P-STAT2, in association with IRF9, form a heterotrimeric complex to enter into the nuclei and drive the ISRE transcription [45]. We hypothesized that the inhibition of STAT1 phosphorylation may result in the block of P-STAT2 nuclear translocation. To verify this hypothesis, we stained empty vector or NS4B transfected cells with the antibody for P-STAT2 and established its subcellular localization by immunofluorescence (Figure 5a).

Results confirmed that, indeed, P-STAT2 nuclear localization was blocked. In fact, in contrast to empty vector transfected cells in which P-STAT2 was only nuclear, in NS4B transfected cells, P-STAT2 was only detected in the cytoplasm (Figure 5a).

To understand if the block of P-STAT2 nuclear translocation occurs concurrently as the inhibition of P-STAT1, we evaluated the P-STAT2 subcellular localization at 24 h or 36 h after NS4B transfection. Results showed that the reduction in P-STAT2 nuclear transport was temporally comparable with the signal reduction in P-STAT1. At 24 h, nuclei immunostaining for P-STAT2 amounted to a proportion of 58%, while at 36 h was only 29% (Figure 5b). Taken together, these data confirm that NS4B was inhibiting P-STAT2 nuclear transport and that this block was due to the indirect inhibition of STAT1 phosphorylation, since both events occur at the same hours post-transfection.

## 4. Discussion

The 2015–2016 ZIKV outbreak in South and Central America, and its association with neurological birth defects, have attracted global attention to the need to identify potential virulence determinants and to develop therapeutic interventions against them. In particular, the novel ZIKV strains have accumulated mutations in residues involved in anti-host countermeasures that promote the viral antagonism of the innate response, resulting in increased viral replication and pathogenesis in humans [20]. Interestingly, amino acid changes were found also in viral protein NS4B, highlighting the importance of NS4B as a determinant of viral pathogenesis [46].

In this study, we reported the characterization of NS4B of a novel strain of ZIKV, Brazil/2016/INMI1, with its contribution to the IFN signaling antagonism. Previous studies have shown that ZIKV INMI1 strain infection does not elicit a strong type I-III IFN responses and poorly activates type II IFNs [22]. The ability of ZIKV to evade the IFN response has been previously demonstrated [47,48]. In particular, three proteins encoded by ZIKV have been already identified as inhibitors of the JAK/STAT cascade: NS2A, NS2B3 and NS5 [19,21,23,49,50]. We report that INMI1 NS4B also blocks the IFN-I/-II signaling cascades, dose-dependently inhibiting the ISRE and GAS transcription and the mRNA expression levels of *ISG15* and *OAS1*.

We previously reported the ability of INMI1 NS2A to evade the IFN signaling [23]. The synergistic effect of NS2A and NS4B suggested that the two proteins may counteract the same step of the cascade, acting with a different mode and/or may counteract different steps. We observed that NS4B is able to inhibit STAT1 phosphorylation. Our previous results showed that STAT1 phosphorylation was also suppressed in presence of NS2A [23]. However, the mechanism of action of the two proteins is different. In fact, the inhibition of STAT1 phosphorylation, induced by NS2A, is the consequence of the degradation of STAT1 by the viral protein. In contrast, NS4B does not seem to act through STAT1 degradation, but directly affects its phosphorylation. Moreover, the stronger effect of NS2A in inhibiting the ISRE expression may be due to the fact that NS2A also interferes at the level of STAT2, by mediating its degradation [23].

The NS4B inhibition of STAT1 phosphorylation has been demonstrated also in other flaviviruses, including DENV. Our analysis showed that the portion NS4B_77-125_, known as the IFN inhibitory domain in DEN2, was highly conserved between DEN2 and INMI1 NS4B. In addition, the INMI1 NS4B predicted topology resulted to be similar to that of DENV, containing three transmembrane segments at the N-terminal portion of the protein. Experimental structural studies on the full-length NS4B and, in particular, on the portion NS4B_77-125_ will provide more information to unravel its ER lumen/cytoplasmic orientation and its contribution to the IFN evasion. This will be helpful in understanding the interaction with cellular targets upstream to the step of STAT1 phosphorylation. The analysis of the ZIKV-human interactome revealed potential interactions between NS4B and JAK1 and STAT1 with PPI scores of 0.4 and 0.6, respectively [51]. Thus, it is likely that NS4B could potentially interact with these two proteins. However, experimental studies are necessary to unravel the mechanistic insight of the NS4B IFN evasion. It is attractive to understand how an ER-localized protein may interact with the cytoplasmic proteins involved in the JAK/STAT cascade, which are mainly localized nearby or in the plasma membrane.

Another interesting consideration to address is the potential role of the 2K peptide in the ability of NS4B to inhibit the IFN signaling. The 2K signal peptide consists of 23 amino acids and is located between the C-terminus of NS4A and the N-terminus of NS4B (NS4A-2K-NS4B). During infection, the viral serine protease NS2B3 first cleaves the NS4A/2K-NS4B junction, forming fragment 2K-NS4B, that is translocated into the ER lumen. The host signalase then cleaves the 2K peptide, generating the amino-terminal end of NS4B. Transfection studies have demonstrated that partial cleavage of the 2K segment by the host peptidase occurs in DEN2 and YFV NS4B proteins, while a more efficient cleavage occurs in WNV 2K-NS4B [16,52]. It has been demonstrated that the cleavage is dependent on SPCS1 and other members of the signal peptide processing pathway. The loss of SPCS1 expression also negatively affects ZIKV titers, suggesting that the same protease complex is critical also for ZIV infection [52]. Despite the fact that a detailed cleavage mechanism has not been investigated for ZIKV 2K-NS4B, our experiments suggest that the cleavage is occurring also in the case of ZIKV 2K-NS4B. In fact, the NS4B immunoprecipitation results in the recovery of two bands, one corresponding to the size of 2K-NS4B (upper band) and the other one corresponding to the size of NS4B alone (Figure S3). The cleavage is only partial, in fact, most of the protein is in the form 2K-NS4B. In DENV, the 2K segment of NS4B is not directly involved in the NS4B IFN inhibitory function since its substitution with the murine MHC-I signal peptide KB resulted in an equally functional protein [16]. However, the lack of either signal peptide resulted in a non-functional NS4B protein, suggesting that these amino-terminal sequences are important for the correct ER localization of NS4B. Given the high similarity between DEN and ZIKV 2K-NS4B sequences, we cannot exclude that the same considerations are also valid for ZIKV. The fact that the main form of ZIKV 2K-NS4B is the not cleaved, may suggest that this is also functionally active, even if we cannot ascertain whether both 2K-peptide-containing and 2K-peptide-cleaved NS4B proteins are functional inhibitors of IFN signaling.

The majority of viruses, during their evolution, have evolved several strategies for counteracting the host defense mechanisms [53,54,55,56]. Many antiviral drugs are designed to target the earliest points of a viral infection, such as entry and fusion [57,58,59]. After entry, one of the first steps in the cellular anti-viral response is the IFN system activation. Hence, a potent antiviral approach is the restoration of the host IFN response to block the infection early. This can be achieved by directly targeting viral IFN antagonists [28,60,61,62,63,64,65] and/or enhancing the cellular innate immunity [66,67,68,69]. NS4B is a well-investigated target for flaviviral therapy [70]. Different phenotypic cell-based assays screening for flavivirus inhibitors resulted in the identification of molecules targeting NS4B [70]. Small molecule inhibitors of HCV and DENV NS4B have been discovered, and it is suggested that such inhibitors may suppress HCV or DENV replication, partly by counteracting the ability of NS4B to antagonize the IFN system and partly by restoring the cellular antiviral responses [65,71]. Given the implications of NS4B IFN antagonism for ZIKV pathology, this protein can be exploited as therapeutic target to aid an immune response against ZIKV.

In conclusion, this study unravels a novel ZIKV-host interaction that helps to elucidate its mechanisms of IFN antagonism and may constitute a potential target to block the infection.

## Figures and Tables

**Table 1:** Primers for subcloning.

Primer Name ^1^	Sequence (5′ to 3′)
2K-NS4B_F	TCTCCCCAGGACAACCAAATG
2K-NS4B_R	ACGTCTCTTGACCAAGCCAGC

^1^ F, forward; R, reverse.

**Table 2:** Primers for RT-qPCR.

Primer Name ^1^	Sequence (5′ to 3′)
*GAPDH_F*	GAGTCAACGGATTTTGGTCGT
*GAPDH_R*	TTGATTTTGGAGGGATCTCG
*ISG15_F*	TCCTGGTGAGGAATAACAAGGG
*ISG15_R*	CTCAGCCAGAACAGGTCGTC
*OAS1_F*	AGCTTCATGGAGAGGGGCA
*OAS1_R*	AGGCCTGGCTGAATTACCCAT

^1^ F, forward; R, reverse.

**Table 3:** NS2A and NS4B combination assay.

Protein	EC_50_ (ng/well)	FIC_50_ A/B ^1^	FIC_50_ ^1^	Combination Effect
NS2A (A)	40.15	0.35	0.5	Synergism
NS4B (B)	93	0.15		

^1^ FIC_50_ values were determined according to the fixed-ratio method of Fivelman et al. [27]. FIC_50_ < 1—synergistic action; FIC_50_ = 1—additive action; FIC_50_ > 1—antagonistic action [26].

## Supplementary Materials

The following are available online at https://www.mdpi.com/article/10.3390/v13122448/s1, Figure S1: Amino acid alignment of INMI1 KU991811 NS4B and other ZIKV strains with percentage identity matrix, Figure S2: Phylogenic analysis of NS4B, Figure S3: Immunoprecipitation of FLAG-tagged NS2A and NS4B.
