# Supplementary material for: INMI1 Zika Virus NS4B Antagonizes the Interferon Signaling by Suppressing STAT1 Phosphorylation
Source: Viruses. 2021 Dec 6;13(12):2448. doi: 10.3390/v13122448 (PMC8705506; doi:10.3390/v13122448)
Supplement: Supplementary file 1 [file viruses-13-02448-s001.zip › viruses-1463576-supplementary.pdf]

## SUPPLEMENTARY FILES

**Fanunza at al. 2021. INMI1 Zika Virus NS4B Antagonizes the Interferon Signaling by suppressing STAT1 phosphorylation**

**(a)**

```
INMI1           NELGWLERTKSDLSHLMGRREEGATIGFSMDIDLRPASAWAIYAALTTFITPAVQHAVTT   60
FSS13025        NELGWLERTKSDLSHLMGRREEGATIGFSMDIDLRPASAWAIYAALTTFITPAVQHAVTT   60
Z1106033        NELGWLERTKSDLSHLMGRREEGATIGFSMDIDLRPASAWAIYAALTTFITPAVQHAVTT   60
PRVABC-59       NELGWLERTKSDLSHLMGRREEGATIGFSMDIDLRPASAWAIYAALTTFITPAVQHAVTT   60
10087PF         NELGWLERTKSDLSHLMGRREEGATIGFSMDIDLRPASAWAIYAALTTFITPAVQHAVTT   60
Dakar-41525     NELGWLERTKSDIAHLMGRKEEGTTMGFSMDIDLRPASAWAIYAALTTLITPAVQHAVTT   60
MR766           NELGWLERTKNDIAHLMGRREEGATMGFSMDIDLRPASAWAIYAALTTLITPAVQHAVTT   60
                **********.*::*****:***:*:**********************:***********

INMI1           SYNNYSLMAMATQAGVLFGMGKGMPFYAWDFGVPLLMIGCYSQLTPLTLIVAIILLVAHY   120
FSS13025        SYNNYSLMAMATQAGVLFGMGKGMPFYAWDFGVPLLMIGCYSQLTPLTLIVAIILLVAHY   120
Z1106033        SYNNYSLMAMATQAGVLFGMGKGMPFYAWDFGVPLLMIGCYSQLTPLTLIVAIILLVAHY   120
PRVABC-59       SYNNYSLMAMATQAGVLFGMGKGMPFYAWDFGVPLLMIGCYSQLTPLTLIVAIILLVAHY   120
10087PF         SYNNYSLMAMATQAGVLFGMGKGMPFYAWDFGVPLLMIGCYSQLTPLTLIVAIILLVAHY   120
Dakar-41525     SYNNYSLMAMATQAGVLFGMGKGMPFYAWDFGVPLLMMGCYSQLTPLTLIVAIILLVAHY   120
MR766           SYNNYSLMAMATQAGVLFGMGKGMPFYAWDLGVPLLMMGCYSQLTPLTLIVAIILLVAHY   120
                ******************************:******:**********************

INMI1           MYLIPGLQAAAARAAQKRTAAGIMKNPVVDGIVVTDIDTMTIDPQVEKKMGQVLLIAVAV   180
FSS13025        MYLIPGLQAAAARAAQKRTAAGIMKNPVVDGIVVTDIDTMTIDPQVEKKMGQVLLIAVAV   180
Z1106033        MYLIPGLQAAAARAAQKRTAAGIMKNPVVDGIVVTDIDTMTIDPQVEKKMGQVLLIAVAV   180
PRVABC-59       MYLIPGLQAAAARAAQKRTAAGIMKNPVVDGIVVTDIDTMTIDPQVEKKMGQVLLIAVAV   180
10087PF         MYLIPGLQAAAARAAQKRTAAGIMKNPVVDGIVVTDIDTMTIDPQVEKKMGQVLLIAVAV   180
Dakar-41525     MYLIPGLQAAAARAAQKRTAAGIMKNPVVDGIVVTDIDTMTIDPQVEKKMGQVLLIAVAV   180
MR766           MYLIPGLQAAAARAAQKRTAAGIMKNPVVDGIVVTDIDTMTIDPQVEKKMGQVLLIAVAI   180
                ***********************************************************:

INMI1           SSAILSRTAWGWGEAGALITAATSTLWEGSPNKYWNSSTATSLCNIFRGSYLAGASLIYT   240
FSS13025        SSAILSRTAWGWGEAGALITAATSTLWEGSPNKYWNSSTATSLCNIFRGSYLAGASLIYT   240
Z1106033        SSAILSRTAWGWGEAGALITAATSTLWEGSPNKYWNSSTATSLCNIFRGSYLAGASLIYT   240
PRVABC-59       SSAILSRTAWGWGEAGALITAATSTLWEGSPNKYWNSSTATSLCNIFRGSYLAGASLIYT   240
10087PF         SSAILSRTAWGWGEAGALITAATSTLWEGSPNKYWNSSTATSLCNIFRGSYLAGASLIYT   240
Dakar-41525     SSAVLLRTAWGWGEAGALITAATSTLWEGSPNKYWNSSTATSLCNIFRGSYLAGASLIYT   240
MR766           SSAVLLRTAWGWGEAGALITAATSTLWEGSPNKYWNSSTATSLCNIFRGSYLAGASLIYT   240
                ***:* ******************************************************

INMI1           VTRNAGLVKRR    251
FSS13025        VTRNAGLVKRR    251
Z1106033        VTRNAGLVKRR    251
PRVABC-59       VTRNAGLVKRR    251
10087PF         VTRNAGLVKRR    251
Dakar-41525     VTRNAGLVKRR    251
MR766           VTRNAGLVKRR    251
                ***********
```

**(b)**

| | | | | | | | |
|---|---|---|---|---|---|---|---|
| 1: INMI1 | 100.00 | 100.00 | 100.00 | 100.00 | 100.00 | 96.41 | 96.02 |
| 2: FSS13025 | 100.00 | 100.00 | 100.00 | 100.00 | 100.00 | 96.41 | 96.02 |
| 3: Z1106033 | 100.00 | 100.00 | 100.00 | 100.00 | 100.00 | 96.41 | 96.02 |
| 4: PRVABC-59 | 100.00 | 100.00 | 100.00 | 100.00 | 100.00 | 96.41 | 96.02 |
| 5: 10087PF | 100.00 | 100.00 | 100.00 | 100.00 | 100.00 | 96.41 | 96.02 |
| 6: Dakar-41525 | 96.41 | 96.41 | 96.41 | 96.41 | 96.41 | 100.00 | 98.01 |
| 7: MR766 | 96.02 | 96.02 | 96.02 | 96.02 | 96.02 | 98.01 | 100.00 |

**Figure S1.** (**a**) Amino acid alignment of INMI1 KU991811 NS4B and other ZIKV strains with (**b**) percentage identity matrix. Amino acid sequence aligned were INMI1 (GenBank KU991811.1), FSS13025 (Uniprot ZIKVK A0A142I5B9), Z1106033 (GenBank KU312312.1), PRVABC-59 (Gen Bank KX377337.1), Dakar-41525 (GenBank MG758786.1), MR766 (Uniprot ZIKV sp|Q32ZE1|), 10087PF (Uniprot ZIKVF sp|A0A024B7W1|).

(a)

| Viral strain | Identity, % |
|---|---|
| DEN26 sp\|P29990\| | 52.4 |
| DEN27 sp\|P29991 | 52.4 |
| DEN2N sp\|P14340\| | 53.3 |
| DEN28 sp\|P14337\| | 53.3 |
| DEN2J sp\|P07564\| | 52.9 |
| DEN2P sp\|P12823\| | 53.7 |
| DEN2Q sp\|Q9WDA6\| | 53.3 |
| DEN4H sp\|Q2YHF2\| | 49.6 |
| DEN4P sp\|Q58HT7\| | 50.0 |
| DEN4T sp\|Q2YHF0\| | 49.6 |
| DEN4D sp\|P09866\| | 49.6 |
| DEN4S sp\|Q5UCB8\| | 49.6 |
| DEN1S sp\|P33478\| | 51.4 |
| DEN1W sp\|P17763\| | 51.0 |
| DEN1B sp\|P27909\| | 51.0 |
| DEN3M sp\|Q6YMS3\| | 51.6 |
| DEN3S sp\|Q6YMS4\| | 51.6 |
| DEN3I sp\|Q5UB51\| | 50.8 |
| ZIKV KU991811.1 | 100.0 |
| ZIKVF sp\|A0A024B7W1\| | 100.0 |
| ZIKV sp\|Q32ZE1\| | 94.8 |
| SPOV\|A0A1I9RZV1\| | 82.1 |
| SPOV\|A0A1I9RZV2\| | 82.1 |
| SPOV\|A0A2L1GGB4\| | 81.3 |
| SPOV \|C8XPB6\| | 82.1 |
| KUNV sp\|P14335\| | 43.0 |
| WNV9 sp\|Q9Q6P4\| | 43.4 |
| WNV sp\|P06935\| | 42.4 |
| USUV sp\|Q5WPU5\| | 45.4 |
| JEV1 sp\|P27395\| | 47.2 |
| JEV5 sp\|P19110\| | 47.2 |
| JEVJ sp\|P32886\| | 46.8 |
| YFVU sp\|Q1X880\| | 32.9 |
| YFVE sp\|Q074N0\| | 33.3 |
| YFVN sp\|Q1X881\| | 33.7 |
| YFVT sp\|Q9YRV3\| | 33.5 |
| YFVF sp\|Q89277\| | 33.5 |
| YFVI sp\|Q98803\| | 33.1 |
| YFVC sp\|Q6J3P1\| | 33.1 |
| YFV1 sp\|P03314\| | 32.2 |
| YFVA sp\|Q6DV88\| | 33.1 |
| POWVL sp\|Q04538\| | 27.1 |
| LGTV LP21 sp\|P29837\| | 27.1 |
| TBEVS sp\|P07720\| | 26.0 |
| TBEVH sp\|Q01299\| | 27.1 |
| TBEVW sp\|P14336\| | 27.1 |

(b)

| Viral strain | Identity, % |
|---|---|
| ZIKV KU991811.1 | 100.0 |
| DEN2P sp\|P12823\| | 53.7 |
| DEN26 sp\|P29990\| | 52.9 |
| DEN27 sp\|P29991 | 52.9 |
| DEN2Q sp\|Q9WDA6\| | 52.9 |
| DEN2N sp\|P14340\| | 52.9 |
| DEN28 sp\|P14337\| | 52.9 |
| DEN2J sp\|P07564\| | 52.0 |
| DEN1S sp\|P33478\| | 49.2 |
| DEN3M sp\|Q6YMS3\| | 48.8 |
| DEN3S sp\|Q6YMS4\| | 48.8 |
| DEN3I sp\|Q5UB51\| | 48.8 |
| DEN1W sp\|P17763\| | 48.4 |
| DEN1B sp\|P27909\| | 48.4 |
| DEN4P sp\|Q58HT7\| | 47.5 |
| DEN4S sp\|Q5UCB8\| | 46.7 |
| DEN4T sp\|Q2YHF0\| | 46.7 |
| DEN4H sp\|Q2YHF2\| | 46.7 |
| DEN4D sp\|P09866\| | 46.7 |

**Figure S2.** Phylogenic analysis of NS4B. Amino acid identity matrix of (**a**) entire NS4B protein and (**b**) partial $NS4B_{1-125}$.

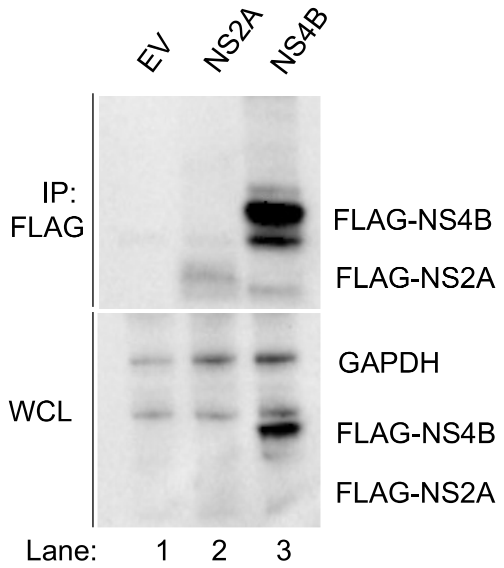


**Figure S3.** Immunoprecipitation of FLAG-tagged NS2A and NS4B using anti-FLAG magnetic beads (IP:FLAG); immunoprecipitated proteins were detected by Western blot with antibodies to FLAG. The presence of transfected protein in whole cell extracts (WCE) was verified by detection with the corresponding antibody.
